# Functional biomarkers complementing static BRCA/HRD testing for dynamic PARP inhibitor therapy in ovarian cancer: a narrative review

**DOI:** 10.3389/fonc.2026.1989686

**Published:** 2026-10-13

**Authors:** Tao Wu, Pengchuang Zhang, Ruxing Xi, Xiaowei Wang, Lijuan Hu, Yahui Shen, Kai Tao, Jianrong Lu, Guoqing Wang

**Affiliations:** 1 Department of Gynecologic Cancer, Shaanxi Provincial Cancer Hospital, Xi’an, Shaanxi, China; 2 Department of Pathology, Shaanxi Provincial Cancer Hospital, Xi’an, Shaanxi, China

**Keywords:** circulating tumor DNA, functional biomarker, KELIM, minimal residual disease, ovarian cancer, PARP inhibitor, RAD51

## Abstract

Poly (ADP-ribose) polymerase inhibitors (PARPi) have revolutionized the management of high-grade serous ovarian cancer (HGSOC). Yet, current treatment decisions remain almost exclusively guided by static BRCA/homologous recombination deficiency (HRD) genotyping. This paradigm is increasingly challenged by a profound eligibility–benefit gap: real-world data show 93.4% of patients are PARPi-eligible, but only 22.0% achieve meaningful 2-year progression-free survival benefit, compounded by 10–30% HRD test failure rates, inability to capture dynamic resistance evolution, and negligible predictive value in the ~50% of HGSOC that are homologous recombination-proficient. This narrative review, based on literature up to June 2026, systematically evaluates functional biomarkers that complement BRCA/HRD testing by assessing real-time tumor biology. To our knowledge, this is the first review to integrate these biomarkers into a unified whole-disease-course framework accounting for evidence level, assay cost, and global resource setting. RAD51 immunohistochemistry directly visualizes homologous recombination repair capacity, achieving 95% accuracy in predicting PARPi response in preclinical patient-derived xenograft models and demonstrating robust predictive value in prospective clinical cohorts. The KELIM model, derived from early CA-125 kinetics and evaluated in >12,000 patients within prospective trial and registry datasets, provides a no-additional-assay-cost, tissue-independent readout of chemosensitivity with potential for broad, low-cost global deployment. Circulating tumor DNA enables noninvasive longitudinal monitoring of resistance, potentially preceding radiographic progression by weeks to months. Exploratory immune and artificial intelligence-based multimodal markers further refine response stratification. We propose deployment of these biomarkers at four critical clinical nodes—diagnosis, post-chemotherapy, maintenance, and progression—to guide therapy selection dynamically. By transitioning from static genotype-based eligibility to real-time functional phenotyping, this integrated strategy aims to close the eligibility–benefit gap and advance adaptive precision oncology for ovarian cancer globally. As a narrative review, this work aims to offer forward-looking conceptual frameworks and stimulate future research rather than to establish high-level clinical evidence or formal guidelines.

## 1 Introduction

The clinical introduction of poly (ADP-ribose) polymerase inhibitors (PARPi) has reshaped the therapeutic landscape of high-grade serous ovarian cancer (HGSOC), delivering unprecedented progression-free survival (PFS) gains in patients with BRCA1/2 mutations or homologous recombination deficiency (HRD) (1–3). Consequently, BRCA/HRD testing has become an indispensable standard-of-care component for newly diagnosed advanced-stage disease (4). However, as clinical experience has matured, BRCA/HRD testing as a static genotypic biomarker has proven increasingly inadequate as the sole basis for clinical decision-making. The most compelling evidence is the disconnect between eligibility and benefit. Carballo et al. (5) showed that by 2021, 93.4% of U.S. epithelial ovarian cancer patients were eligible for PARPi, yet only 22.0% achieved meaningful 2-year PFS benefit. This profound eligibility–benefit gap demands reappraisal of BRCA/HRD testing as a predictive biomarker.

This inadequacy stems from three interrelated limitations. First, technical and tissue-related barriers hamper HRD testing in real-world practice. Across multiple cohorts, HRD test failure rates range from 10% to 30%—substantially higher than in clinical trials (6, 7) with success declining from 91.5% in primary surgery specimens to 51.2% in post-NACT samples (7). Inter-assay positive agreement is only 75–80% (8, 9). Second, static genotyping captures only a biological snapshot at initial surgery and cannot track clonal evolution under therapy. BRCA reversion mutations are detected in 26.0% of patients progressing after PARPi or platinum therapy (10), and RAD51C promoter methylation loss drives resistance under treatment pressure (11). A single biopsy cannot predict functional recovery under sustained drug exposure. Third, HR-proficient (HRP) patients, representing ~50% of HGSOC, derive limited predictive value from BRCA/HRD testing (12): in a real-world cohort, HRP patients on PARPi maintenance had shorter PFS than those not receiving PARPi (12.7 vs. 15.2 months) (13).

To be clear, these limitations do not diminish the foundational role of BRCA/HRD testing, which remains the indispensable entry point for molecular classification of ovarian cancer and has enabled the clinical development of PARP inhibitors. Rather, they underscore that BRCA/HRD status is necessary but no longer sufficient—it must be complemented by functional biomarkers that capture the dynamic nature of homologous recombination competence. BRCA/HRD testing captures genotype but not current functional repair capacity, which is dynamically regulated by epigenetic modifications (10), the immune microenvironment and cytokine signaling (14), and clonal selection from prior therapies (15). A paradigm shift from static genotype to dynamic functional phenotype is therefore needed, and recent functional biomarkers offer practical tools to operationalize this shift: RAD51 foci by immunohistochemistry directly assess HR repair (16–18), KELIM models chemosensitivity from CA-125 kinetics (19–21), and ctDNA tracks resistance evolution (22, 23).

To date, no review has systematically integrated these emerging functional biomarkers into a unified, disease-course-spanning clinical decision framework that accounts for evidence level, assay accessibility, and resource setting. This narrative review evaluates the clinical evidence for these biomarkers, stratifies them by clinical readiness, and proposes a clinically actionable whole-disease-course framework for integrating them into PARPi therapy decisions from diagnosis through progression, narrowing the eligibility-benefit gap in contemporary ovarian cancer care. Accordingly, our primary objective is to offer forward-looking conceptual frameworks and stimulate future research, rather than to establish high-level clinical evidence or formal practice guidelines.

## 2 Methods

### 2.1 Search strategy

A targeted literature search was performed in PubMed, Embase, and Web of Science from database inception to June 20, 2026. The search strategy combined Medical Subject Headings (MeSH) and free-text terms across three domains (1): ovarian cancer terms (“ovarian neoplasm”, “ovarian cancer”, “high-grade serous ovarian cancer”, “HGSOC”); (2) PARP inhibitor and biomarker terms (“PARP inhibitor”, “olaparib”, “niraparib”, “rucaparib”, “talazoparib”, “veliparib”, “RAD51”, “KELIM”, “circulating tumor DNA”, “ctDNA”, “homologous recombination deficiency”, “HRD”, “T cell receptor”, “TCR”, “tumor microenvironment”, “artificial intelligence”, “deep learning”); and (3) precision medicine terms (“predictive biomarker”, “prognostic biomarker”, “precision medicine”, “treatment resistance”, “clinical decision-making”). We used Boolean operators (AND/OR) to link terms within and across domains. Only English-language full-text articles were considered. Detailed reproducible search strategies for PubMed, Embase, and Web of Science are provided in the Supplementary Materials.

### 2.2 Study selection and evidence grading

Eligible studies included: (1) clinical studies evaluating functional biomarker associations with PARP inhibitor efficacy or resistance in ovarian cancer; (2) translational studies informing clinical decision-making; and (3) high-quality systematic reviews and meta-analyses on relevant biomarker categories. Exclusion criteria included: case reports, editorials, conference abstracts without full peer-reviewed data, preclinical studies without clear translational correlates, and studies with sample sizes <20 patients for clinical biomarker analyses. Two authors independently screened titles and abstracts, with disagreements resolved by consensus. Given the narrative nature of this review, we did not perform a formal systematic review methodology or meta-analytic synthesis.

We stratified included biomarkers into three evidence tiers to guide clinical interpretation: Tier 1 (clinically actionable): biomarkers with robust validation in >1000 patients, including prospective or large retrospective cohort data, suitable for routine clinical use; Tier 2 (clinically promising): biomarkers with consistent retrospective validation in multiple independent cohorts, available in reference centers but not yet standard of care; Tier 3 (exploratory): biomarkers with preliminary single-center or preclinical data, limited to research settings at present (12, 24).

## 3 Tissue-based functional homologous recombination assessment

Genomic scar assays for HRD provide a historical record of DNA damage but do not reflect current HR repair capacity. Functional tissue-based assays directly measure tumor cells’ ability to perform homologous recombination in real time, addressing a key limitation of static genotyping.

### 3.1 RAD51 immunohistochemistry—from genotypic “BRCAness” to phenotypic “BRCAness”

The functional integrity of homologous recombination repair (HRR) is reflected in RAD51 activity, the pathway’s core recombinase. The formation of nuclear foci at DNA double-strand break (DSB) sites serves as direct morphological evidence of functional HR repair (25). In contrast to BRCA1/2 gene mutations or genomic scar scores, which reflect “potential” or “historical” HR defects, RAD51 foci assessment evaluates the current HR activation capacity of tumor cells in response to DNA damage stimuli (26). RAD51 foci formation integrates the signaling cascade from damage sensing to effector recruitment, providing a more comprehensive HR assessment than single-gene testing and capturing dynamic treatment-induced changes (27).

Two RAD51 readouts should be distinguished. The functional foci assay counts discrete RAD51 foci at DNA double-strand breaks in geminin-positive (S/G2-phase) cells—reported as foci per nucleus or as the percentage of geminin-positive cells containing ≥N foci; immunofluorescence and brightfield chromogenic immunohistochemistry (the RAD51-FFPE protocol) are interchangeable detection modalities for the same functional readout. The non-foci approach instead scores overall RAD51 nuclear staining intensity (H-score) or the percentage of positive cells across all tumor cells without cell-cycle gating, reflecting protein abundance rather than repair activation.

The clinical predictive value of RAD51 testing has been validated across multiple independent cohorts (**Table 1**). In patient-derived xenograft (PDX) models, when pre-treatment RAD51 functional status was compared with *in vivo* PARPi response as the reference standard, RAD51 testing achieved 95% accuracy (sensitivity 90%, specificity 98%) in predicting PARPi efficacy, compared with 67% accuracy for BRCA1/2/PALB2 mutation status (16). In prospective validation of platinum-based chemotherapy sensitivity, RAD51-Low status—defined as >10% of geminin-positive cells containing <5 RAD51 foci—achieved a positive predictive value of 100% and was significantly associated with prolonged PFS (HR = 0.53) and OS (HR = 0.43) (17), demonstrating robust stratification capacity. Notably, among BRCA-mutant samples, the RAD51-High subgroup exhibited unexpectedly poor platinum response (P = 0.02), indicating that RAD51 testing can identify poor responders within BRCA-mutant tumors that genotyping alone cannot distinguish (26).

**Table 1:** Validation studies of RAD51 foci assessment as a functional homologous recombination deficiency biomarker.

Study (year)	Cohort	Foci scoring cutoff	Key performance metrics	Key findings	Evidence tier
Pellegrino et al. (16)	103 ovarian cancer PDX models	≥2 foci/cell	PARPi response prediction accuracy 95% (vs 67% for HRR mutations, 71% for genomic HRD)	Preclinical proof-of-concept; RAD51 foci outperform genomic HRD markers for functional HR status	Preclinical/Tier 3
Compadre et al. (17)	HGSOC prospective cohort (n=148)	≥1 foci/cell in ≥50% tumor cells	PPV for platinum/PARPi sensitivity 100%; PFS HR = 0.53 (0.31-0.90); OS HR = 0.43 (0.22-0.83)	First prospective clinical validation; RAD51-low patients derive maximal PARPi maintenance benefit	Clinical/ Tier 2
Blanc-Durand et al. (26)	CHIVA prospective trial (n=155)	RAD51-low defined as ≤10% of GMN-positive tumor cells with ≥5 RAD51 foci	Assay contributive rate 92%; NGS sample availability 77%; RAD51-low (HRD) in 54%; higher overall response rate to neoadjuvant platinum (p=0.04); longer PFS (p=0.02)	RAD51 assay feasible on FFPE; RAD51-low predicts platinum sensitivity and longer PFS; RAD51-high BRCAmut shows poor chemotherapy response	Clinical/ Tier 2
Kim et al. (27)	Paired pre/post-chemotherapy samples (n=34 pairs)	≥10% of geminin-positive cells containing RAD51 foci	44% of patients had RAD51 status conversion post-chemotherapy; persistent RAD51-high patients had worst PFS (p=0.031)	Demonstrates dynamic HR status changes with chemotherapy; post-treatment testing improves prediction	Translational/ Tier 2
van Wijk et al. (28)	Endometrial/ovarian cancer FFPE (n=74)	≥2 foci/cell	Sensitivity for BRCA deficiency 90%; 88% concordance with RECAP HRD assay	Methodological validation; established standardized scoring framework	Methodological/ Tier 2

PDX, patient-derived xenograft; PARPi, poly(ADP-ribose) polymerase inhibitor; HRR, homologous recombination repair; HRD, homologous recombination deficiency; HGSOC, high-grade serous ovarian carcinoma; PPV, positive predictive value; PFS, progression-free survival; OS, overall survival; HR, hazard ratio; NGS, next-generation sequencing; FFPE, formalin-fixed paraffin-embedded; RECAP, REpair CAPacity assay; IHC, immunohistochemistry. GMN, geminin (S/G2 phase marker).

All studies listed use a foci-based readout in geminin-positive (S/G2-phase) cells, detected by immunofluorescence or brightfield immunohistochemistry. Studies scoring overall RAD51 staining intensity (H-score) without foci counting or cell-cycle gating represent a distinct, non-foci expression assay and are therefore not included.

Beyond static prediction, RAD51 testing offers broad sample applicability and dynamic monitoring capacity. The RAD51-FFPE assay enables HR assessment on routine formalin-fixed paraffin-embedded sections, achieving 90% sensitivity for BRCA deficiency detection (28); Longitudinal analysis showed chemotherapy-induced RAD51 conversion in 44% of paired samples, with sustained RAD51-Low predicting the best PFS (27). This highlights a key advantage: RAD51 status can be reassessed at progression, whereas genomic scar scores remain fixed (29, 30).

Existing data suggest that RAD51 status holds substantial value in informing treatment strategy selection. Patients with RAD51-High status (HR-functional, >10% cells positive with ≥5 foci) are likely to derive limited benefit from PARPi monotherapy, even in the presence of BRCA mutations (26), and could be prioritized for evaluation of combination strategies (ATRi, WEE1i, or chemotherapy) within clinical trials (31). Conversely, RAD51-Low status (HR-deficient, ≤10% cells positive or <5 foci) indicates a higher likelihood of response to PARPi monotherapy and significantly enhanced platinum sensitivity (16–18). The dynamic transition from RAD51-Low to RAD51-High during treatment may raise suspicion for acquired resistance, as demonstrated by a non-foci RAD51 IHC study reporting increased RAD51 H-scores upon progression on PARPi (32) and RAD51 status conversion in 44% of paired pre/post-chemotherapy samples (27), whereas sustained RAD51-Low status suggests durable HR deficiency and potentially longer PFS with PARPi maintenance.

Important limitations of RAD51 testing warrant consideration. Currently, scoring thresholds remain heterogeneous (5–10 foci per cell; 10–15% positive cells), and a four-laboratory study found median inter-observer RAD51 score ranges of 13.5–18% (upper quartile up to 32%), greatest in heterogeneous or poorly stained samples and most consequential near HRD thresholds (33). Commercial RAD51 IHC assays are now available in reference laboratories. Still, global standardization of scoring criteria and automated AI-assisted quantification are needed before widespread clinical adoption. From a cost perspective, RAD51 IHC is comparatively inexpensive (approximately US$100–200 per sample) and can be implemented on standard immunohistochemistry infrastructure already present in reference pathology laboratories (34), although trained personnel for foci scoring remain a prerequisite; a comparative cost analysis across biomarkers is provided in Section 9.2. Overall, RAD51 testing represents a Tier 2 clinically promising biomarker that shifts stratification from static genotyping toward dynamic functional phenotyping, with particular value for resolving equivocal HRD results and reassessing HR status at progression.

### 3.2 Beyond the “42 Threshold”: functional HRD scoring

The HRD score currently used in clinical practice is derived from a composite of three genomic scar metrics—loss of heterozygosity (LOH), telomeric allelic imbalance (TAI), and large-scale state transitions (LST)—with a threshold of 42 defining HRD positivity (Myriad myChoice^®^ CDx) (35, 36). However, this approach has limitations. First, thresholds vary across trials (≥42 vs ≥33) and assays (e.g., GIScar), yielding discordant classifications for the same patient (37, 38). Second, as a scar-based metric, HRD-score reflects historical genomic damage rather than current HR functionality; tumors may restore HR through BRCA reversion or epigenetic changes while retaining a positive score, resulting in a discordance between genomic scar status and current HR function (39).

Large-scale analyses have shown that only biallelic BRCA1/2 inactivation strongly correlates with high HRD scores, and fewer than 40% of BRCA1/2-mutant tumors exhibit high GIS, challenging the assumption that any HRR gene mutation equates to HRD (40, 41). Notably, simple chromosomal scar-based scores perform comparably to more complex mutational signature models in predicting platinum sensitivity (42). Growing evidence supports treating the HRD score as a continuous variable: Graves et al. (13) found that, in non-BRCA1/2-mutated ovarian cancer, higher GIS as a continuous measure correlated with improved PFS and OS. For the clinically challenging HRD gray zone (GIS 33–50), which accounts for ~20% of all patients, RAD51 functional testing provides critical resolution: in this subgroup, RAD51-Low patients derive similar PFS benefit from PARPi as HRD-high patients, while RAD51-High patients have outcomes comparable to HRP patients (30). Emerging algorithms refine HRD assessment beyond generic genomic scars. ovaHRDscar, optimized for HGSC-specific allelic imbalance patterns, improves the precision of HRD classification compared with pan-cancer criteria (43). tHRD, based on aberrant transcript usage, reflects functional HR status and outperforms genomic HRD and BRCA1/2 screening in predicting PARPi and platinum response (44). However, these approaches remain investigational because they lack prospective validation, require WGS or RNA-seq platforms not standardized for routine FFPE testing, have no regulatory approval or harmonized cutoffs, and carry substantially higher costs than current targeted HRD assays (45).

Synthesizing this evidence, the proposed framework suggests that GIS values could be integrated with RAD51 functional testing rather than relying on a single 42 threshold (46). We propose a tiered framework: GIS ≥42 (HRD-high) supports PARPi maintenance; GIS 33–42 (gray zone) warrants RAD51 functional testing to distinguish HR deficiency from HR proficiency; and GIS <33 (HRP) suggests limited PARPi benefit. In the gray zone, RAD51-low patients derive PFS benefit similar to HRD-high patients, whereas RAD51-high patients have outcomes comparable to HRP patients (30). This approach is feasible because GIS is already reported as a numerical value by commercial assays, and RAD51 IHC can be performed on the same FFPE tissue. For resource-limited settings where RAD51 is unavailable, the continuous GIS value can still inform individualized risk–benefit discussions. Both approaches risk false positivity and false negativity near the threshold—a dilemma resolvable through RAD51 functional validation (30). Furthermore, given that HRD status is dynamic, repeat functional assessment at key decision points (e.g., recurrence, progression) can inform subsequent therapy selection (47). Prospective validation of this continuous-GIS framework is warranted.

## 4 Non-invasive chemosensitivity assessment: the KELIM model

If RAD51 foci directly visualize the DNA repair behavior of tumor cells under a microscope, then KELIM (CA-125 elimination rate constant) offers an alternative, highly accessible window into tumor biology—one based on mathematical modeling of routinely collected clinical data, and requiring no additional assay beyond standard CA-125 measurements. KELIM is not a novel experimental assay but a computational parameter derived from the longitudinal kinetics of CA-125 during the first 100 days of platinum-based chemotherapy (48, 49). By quantifying the rate of CA-125 clearance, this model provides a dynamic readout of the tumor’s intrinsic chemosensitivity to DNA-damaging agents, independent of BRCA/HRD status.

The clinical utility of KELIM has been evaluated across more than 12,000 patients in multiple independent prospective-trial and registry cohorts. In a large individual-patient meta-analysis (n=5,884), favorable KELIM (≥1.0) was independently associated with prolonged OS (78.8 vs 28.4 months; HR 0.46) (21). In the PARPi setting, Colomban et al. demonstrated in 476 rucaparib-treated patients (ARIEL2/Study 10) that KELIM independently predicted radiological response (OR 2.81) and PFS (HR 0.67), with durable benefit extending to BRCA wild-type and HRP patients (19). KELIM also identifies those most likely to benefit from bevacizumab in the first-line setting (50).

A key advantage of KELIM is its universal accessibility: the calculation requires no additional laboratory assay beyond the CA-125 values already measured during the first three chemotherapy cycles and takes only a few minutes using a freely accessible online tool(https://www.biomarker-kinetics.org/CA-125). This makes KELIM uniquely valuable in resource-limited settings where tissue-based HRD testing or ctDNA analysis is unavailable. A recent national survey indicates that 58% of surveyed French gynecologic oncologists had used KELIM, mainly to assess chemosensitivity and guide bevacizumab use (51). Clinically, KELIM provides a straightforward stratification framework: a favorable score (≥1.0) indicates high sensitivity to DNA-damaging agents, supporting PARPi monotherapy maintenance even in HRP patients; an unfavorable score (<1.0) suggests limited response to platinum and PARP inhibition, favoring combination strategies—including bevacizumab, ATR inhibitors, or dose-dense chemotherapy regimens (52).

Several important caveats warrant emphasis. First, KELIM is non-calculable in approximately 15% of patients owing to insufficient CA-125 measurements within the first 100 days or a normal baseline value; kinetics may be distorted by ascites or peritoneal inflammation, and CA-125 non-secretor tumors are inherently un-assessable, in which cases radiologic and clinical assessment substitutes. Second, KELIM has not undergone regulatory-grade analytical validation: no published precision, accuracy, or inter-laboratory reproducibility studies have used Clinical and Laboratory Standards Institute (CLSI) or equivalent protocols, and the impact of inter-assay CA-125 variability on KELIM classification remains unquantified. Third, the online calculator is an academic research tool, without regulatory clearance as a medical device or clinical decision-support software; formal local software validation would be required before routine deployment. Finally, KELIM is not literally cost-free: although it adds no assay or infrastructure cost, data entry, calculation, and documentation require staff time—a hidden full-time-equivalent staff cost that has not been quantified in health-economic analyses and may matter in resource-limited settings (52). These gaps define the validation work required for regulatory adoption; they do not alter KELIM’s position as the lowest-cost, most widely deployable functional biomarker (Tier 1 reflects the strength of clinical evidence, not regulatory status).

## 5 Liquid biopsy for real-time resistance monitoring

If static BRCA/HRD testing answers “what genotype does the tumor carry at diagnosis”, then dynamic ctDNA monitoring addresses a far more urgent question: “how is the tumor’s repair capacity evolving under therapeutic pressure?” PARP inhibitor resistance is a continuous process of clonal evolution that static tissue biopsies cannot adequately track because of invasiveness and the impracticality of repeated sampling. ctDNA liquid biopsy, by contrast, is non-invasive, serial, and captures the global genomic landscape, making it uniquely suited to fill this monitoring gap. Three complementary ctDNA-based strategies have emerged for tracking resistance in real time: monitoring BRCA reversion mutations, tracking epigenetic marker changes, and serially assessing genomic instability scores.

### 5.1 Tracking BRCA reversion mutations

BRCA reversion mutations represent the best-characterized mechanism of acquired PARP inhibitor resistance. BRCA reversion mutations restore the open reading frame and HR function, thereby escaping PARP inhibitor synthetic lethality (53–55). Because reversion mutations typically emerge in minor subclones, conventional tissue biopsy is ill-suited for their detection; ctDNA, by virtue of its ability to reflect the heterogeneity of the entire tumor burden, offers a distinct advantage in this context (56).

Multiple clinical studies have validated the feasibility and prognostic value of ctDNA-based reversion mutation monitoring. In the EVOLVE trial, Lheureux et al. (22) performed targeted sequencing on 78 longitudinal ctDNA samples from 30 patients with HGSOC who had progressed on PARP inhibitors, detected BRCA1/2 reversion mutations at baseline and demonstrated that differences in mutant fragment size distribution correlated with shorter time to progression. Zong et al., in a large Chinese pan-cancer cohort, found that 58.3% of reversion deletions were flanked by microhomology sequences, implicating microhomology-mediated end-joining as the primary mechanism (57), while Tobalina et al., in a meta-analysis of 327 BRCA-mutant tumors, showed that reversion mutations are more common in BRCA2 than BRCA1 and that most of exon 11 is dispensable for maintaining resistance (10).

From a clinical decision-making perspective, ctDNA-based reversion monitoring has value on two levels. Detection of reversion mutations suggests pre-existing resistant subclones and warrants consideration of combination strategies. During ongoing treatment, a dynamic rise in reversion mutation allele frequency often heralds impending radiographic progression, providing a lead time of weeks to months for early intervention. However, sensitivity is constrained by ctDNA tumor fraction; a negative result should not be interpreted as the absence of resistance risk (58).

### 5.2 Dynamic changes in epigenetic markers: the reversible methylation switch

Hypermethylation of CpG islands in the BRCA1 and RAD51C promoters represents an important epigenetic mechanism of homologous recombination deficiency, functionally equivalent to gene mutations. Unlike genetic mutations, however, DNA methylation is reversible—a property that makes it both a valuable HRD marker and a potential source of acquired resistance under therapeutic pressure (59).

Nesic et al., using PDX models of ovarian cancer, provided the first systematic demonstration of this dynamic process: homozygous RAD51C promoter methylation correlated with gene silencing and HRD phenotype, and PDX models were sensitive to PARP inhibitors; however, during treatment, models acquired loss of RAD51C methylation, with restoration of a single unmethylated allele sufficient to drive PARP inhibitor resistance (11). Blanc-Durand et al., in 100 patients with high-grade ovarian cancer, quantified the clinical significance of BRCA1 methylation, showing that high-level BRCA1 methylation (≥70% threshold) was present in 19% of BRCA wild-type tumors and that all highly methylated tumors were classified as HRD-positive (GIS ≥42), yielding a positive predictive value of 100% (60). On the technical front, Fink et al. established a validated clinical workflow for quantitative BRCA1 and RAD51C promoter methylation testing, demonstrating accurate quantification even in low-input or highly degraded FFPE samples (61).

Collectively, these findings indicate that methylation status can evolve dynamically under treatment pressure. A decline in methylation levels—particularly the transition from homozygous to heterozygous methylation—in patients with baseline methylation positivity should be interpreted as a potential warning signal of emerging resistance. Given the reversibility of methylation, combining demethylating agents with PARP inhibitors may prevent epigenetically driven resistance—a hypothesis warranting future study. Methylation testing is currently available in specialized reference laboratories as a Tier 2 biomarker.

### 5.3 cfDNA-based genomic instability score

The cfDNA-based genomic instability score (GIS) offers a tissue-free, repeatable approach to HRD assessment, valuable when tissue is unavailable or serial monitoring is required (62). Its core advantage is repeatability, making it particularly valuable for patients with unavailable fresh tissue, failed biopsies, or the need for serial monitoring.

Senturk Kirmizitas et al. found that baseline epigenetic differences distinguished resistant from responsive patients, while genomic alterations emerged predominantly after progression (63). Zelisse et al. reported that combined TP53 and copy number analysis improved ctDNA detection, though sensitivity declined substantially after neoadjuvant chemotherapy (64). cfDNA-based GIS has unique value in the following scenarios: patients in whom tissue biopsy is infeasible, those requiring dynamic monitoring of HRD status evolution, and those experiencing “biochemical progression” (rising CA-125 with radiographically stable disease) during PARP inhibitor therapy. However, its limitations warrant attention—unreliability at low tumor fractions, discordance in GIS thresholds across different platforms, and the marked impact of prior chemotherapy on sensitivity. Concordance between tissue and cfDNA GIS is approximately 75–80% in treatment-naïve patients but drops to 50–60% post-chemotherapy due to reduced tumor fraction and clonal hematopoiesis interference (65, 66).

In summary, these three ctDNA strategies are complementary: reversion tracking detects genetic resistance, methylation monitoring captures earlier epigenetic changes, and cfDNA GIS provides a tissue-free HRD snapshot. Their shared value lies in enabling real-time, longitudinal decision support. Integration with RAD51 IHC and KELIM will be essential for dynamically adaptive precision medicine. **Table 2** summarizes the clinical applications and limitations of cfDNA-based GIS, alongside the other two ctDNA strategies.

**Table 2:** Circulating biomarker assays for PARP inhibitor therapy personalization.

Assay	Sample type	Analytical performance	Clinical application	Key limitations	Evidence tier
BRCA reversion mutation testing (22, 39)	Plasma ctDNA	Sensitivity 75-85% for known reversion mutations; specificity >99%	Confirm PARPi resistance at progression; guide selection of subsequent combination therapy	False negatives in low tumor fraction samples; cannot detect non-reversion HR restoration mechanisms	Clinical/Tier 2
BRCA1/RAD51C promoter methylation testing (11, 26, 43)	Plasma cfDNA	Concordance with tissue methylation 82-88%; sensitivity 78% for epigenetic HRD	Early detection of reversible HR restoration; guide demethylating agent combination trials	Methylation levels are dynamic with treatment; requires strict bisulfite conversion controls.	Translational/Tier 3
Plasma genomic instability score (GIS) (63, 64)	Plasma cfDNA	Concordance with tissue GIS 76-83%; AUC 0.81 for HRD classification	Tissue-free HRD assessment when archival tissue is unavailable; serial HRD monitoring	Lower sensitivity in samples with <1% VAF; does not capture functional HR restoration	Translational/Tier 3
ctDNA MRD monitoring (66, 67)	Serial plasma samples	Sensitivity ~80% for histologically confirmed MRD; specificity >95%	Pre-maintenance risk stratification; early PARPi resistance detection (median 4 months pre-radiographic progression)	False negatives in < 5 mm peritoneal disease or isolated CNS metastases; CHIP-related false positives require tumor-informed assay design	Emerging/Tier 2

ctDNA, circulating tumor DNA; cfDNA, cell-free DNA; HRD, homologous recombination deficiency; HR, homologous recombination; PARPi, poly(ADP-ribose) polymerase inhibitor; AUC, area under the curve; VAF, variant allele frequency; MRD, minimal residual disease; CHIP, clonal hematopoiesis of indeterminate potential; CNS, central nervous system; GIS, genomic instability score.

### 5.4 Circulating tumor DNA for minimal residual disease monitoring

The highest-impact emerging application of ctDNA in PARPi therapy is minimal residual disease (MRD) assessment immediately before maintenance initiation and longitudinal monitoring during PARPi treatment, as it directly addresses the core clinical question of which patients derive meaningful benefit from PARPi, and who will develop resistance before radiographic progression.

At the PARPi initiation decision point (post-first-line chemotherapy), detectable ctDNA identifies patients at highest risk of recurrence who derive the greatest absolute benefit from PARPi maintenance. In a multicenter real-world study using a tumor-informed ctDNA assay (Signatera™) in 53 patients on PARPi maintenance, ctDNA was detectable before treatment in 7 of 12 patients (58%) with available pre-PARPi samples; none of these seven achieved sustained clearance, whereas none of the five ctDNA-negative patients recurred at last follow-up, and ctDNA positivity within the first 3 months predicted shorter PFS (p=0.01) (67). In histologically validated MRD cohorts (secondary-look laparotomy, SLL, gold standard) of patients in complete clinical remission after first-line therapy, 42.1% had surgically confirmed residual disease, and ctDNA detected 34% of these MRD cases; ctDNA positivity was associated with a 4.4-fold increased risk of death (median PFS 6.4 vs. 28.1 months; P < 0.001) independent of BRCA/HRD status (68).

During PARPi maintenance, longitudinal ctDNA dynamics provide early evidence of PARPi response or resistance: ctDNA clearance within the first 3 months of PARPi initiation is associated with significantly improved PFS (p=0.003), while persistent ctDNA positivity or conversion to positivity within the first 3 months is associated with significantly shorter PFS (p=0.01) (67). Rising ctDNA variant allele fraction precedes radiographic progression by a median of 4 months, including cases with normal CA-125 and negative imaging at the time of molecular PARPi resistance; ctDNA status is a significantly stronger predictor of PFS than CA-125, BRCA, or HRD status in patients on PARPi maintenance (68).

Limitations include ~80% sensitivity for histologic MRD, with false negatives in < 5 mm peritoneal disease or isolated CNS metastases, and clonal hematopoiesis-related false positives requiring tumor-informed assay design. Multiple prospective and phase 2 studies are currently validating ctDNA MRD-guided PARPi personalization, including trials evaluating ctDNA dynamics to guide maintenance duration, early therapy escalation in molecular progressors, and PARPi de-escalation in persistently ctDNA-negative patients (e.g., NCT07059676, NCT07670962, NCT05065021).

### 5.5 Barriers to routine clinical implementation

ctDNA remains investigational because it lacks prospective validation, standardized assays, regulatory approval, and cost-effectiveness data (69). Cost is a critical barrier. Tumor-informed ctDNA MRD assays cost approximately US$3,000–5,000 per test, and serial monitoring every 3–6 months multiplies this cost per patient, rendering longitudinal profiling cost-prohibitive in routine practice (70). This financial burden extends to pharmaceutical-sponsored serial-sampling studies, where ctDNA monitoring can substantially constrain trial budgets (71). Dedicated reimbursement pathways and cost-effectiveness evidence remain absent (see Section 9.2 for a comparative cost analysis). In ovarian cancer specifically, anatomical confinement to the peritoneal cavity limits ctDNA entry into the bloodstream, resulting in very low mutant allele fractions in localized disease. Low tumor shedding and clonal hematopoiesis of indeterminate potential (CHIP)-related false positives—particularly involving TP53, the dominant driver in HGSOC—further compromise sensitivity and specificity (72). These barriers must be addressed before ctDNA can be routinely integrated into clinical practice.

## 6 Immune microenvironment biomarkers

The antitumor activity of PARPi is not confined to the synthetic lethality exerted on tumor cells; emerging translational evidence has unveiled a more complex immunomodulatory dimension. PARPi can activate the cGAS-STING pathway, inducing type I interferon production and remodeling the tumor immune microenvironment (TME) to recruit and activate cytotoxic CD8^+^ T cells (14, 73–75). Consequently, host immune status—particularly T-cell clonal diversity and the balance of functional T-cell subsets—may be a critical determinant of PARPi efficacy. All immune biomarkers discussed in this section are currently Tier 3 exploratory biomarkers, with insufficient evidence to support routine clinical use at present.

### 6.1 Peripheral immune metrics: TCR diversity and T-cell subsets

T-cell receptor (TCR) repertoire diversity reflects the host’s capacity to recognize and eliminate tumor neoantigens. In a cohort of patients with HGSOC receiving PARPi maintenance therapy, Shu et al. performed sequencing of the TCRβ chain CDR3 regions in peripheral blood and demonstrated a direct association between dynamic changes in TCR diversity and clinical outcomes. Patients with increased TCR diversity 3 months after PARPi initiation had significantly longer PFS (21.7 vs 7.4 months; HR 0.19) (23). Complementary evidence comes from studies focusing on immunosuppressive T-cell subsets. Regulatory T cells (Tregs) are key negative regulators of antitumor immunity. Higher baseline PD-1^+^ Tregs were associated with shorter PFS on PARPi monotherapy, while their decline after triplet therapy correlated with improved outcomes (76). These findings suggest that PARPi alone may partially suppress Tregs but cannot effectively downregulate PD-1 expression; the addition of anti-PD-1 antibody more potently eliminates these highly immunosuppressive cells, relieving the immune brake and enhancing therapeutic efficacy.

Although high-throughput TCR sequencing remains costly and analytically complex, limiting its adoption as a routine clinical biomarker, it holds substantial value as an exploratory tool for elucidating the interplay between PARPi and the immune system, identifying patient populations most likely to benefit from combination immunotherapy, and enabling patient stratification in clinical trials. As sequencing costs decline and bioinformatics pipelines standardize, dynamic TCR diversity monitoring may shift from research to clinical practice.

### 6.2 *In situ* tumor microenvironment profiling

While peripheral TCR diversity and T-cell subsets reflect systemic immune status, biomarkers within the tumor microenvironment (TME) provide direct, *in-situ* evidence of drug action. The TME composition and functional state critically influence antitumor immune responses and serve as histological correlates of PARPi-induced immune activation.

Among conventional immunohistochemistry (IHC)-detectable markers, high PD-L2 expression was independently associated with poor PFS (HR 1.89) despite increased CD8^+^ infiltration, suggesting an immune evasion mechanism (77). This suggests PD-L2 as a key immune evasion pathway that may undermine PARPi immunostimulation despite CD8^+^ infiltration (78). This finding underscores that, rather than simply assessing the density of immune infiltration, precise identification of functionally active tumor-reactive T-cell subsets within the TME is more clinical relevance. CD137^+^ TILs thus emerge as a candidate biomarker for predicting response to PARPi-based combination immunotherapy.

In summary, TME biomarkers provide tissue-based evidence for understanding the mechanisms of PARPi action and predicting therapeutic efficacy. Future integration of multiplex immunohistochemistry (mIHC) to simultaneously evaluate multiple immune cell subsets (e.g., CD8^+^, CD137^+^, PD-1^+^ Tregs) and their spatial distribution within the TME will enable more precise “immune landscape” predictive models, facilitating individualized decision-making for PARPi-based combination immunotherapy. Notably, the recent finding that multifocal TILs are the key histological feature driving DiaThera’s ETI-based prediction of PARPi benefit (see Section 7.2) independently corroborates that TME-derived immune signals contain predictive information beyond genomic HRD status, reinforcing the rationale for integrating immune contexture into functional biomarker panels.

## 7 Multi-omics and AI-driven multimodal integration

While RAD51 functional assays, KELIM kinetic modeling, and ctDNA-based dynamic monitoring provide “functional snapshots” of PARPi resistance from the perspectives of HR repair activity, drug sensitivity, and clonal evolution, respectively, the integration of multi-omics and artificial intelligence (AI) represents a qualitative leap—from “single-dimensional biomarker testing” toward “panoramic resistance profiling”. (79–82) PARPi resistance emerges from the concerted action of genetic, epigenetic, transcriptomic, and proteomic alterations—complexity that multi-omics integration and AI are positioned to address.

### 7.1 Multi-omics signatures

Transcriptomic biomarker development has evolved from single-gene analyses toward multi-gene composite signatures. Transcriptomic signatures, such as transcriptional HRD (tHRD) based on aberrant DNA repair gene transcript usage, have outperformed genomic HRD testing in predicting PARPi response (44). Single-cell resolution drug response prediction and proteomic classifiers (e.g., random forest model AUC 0.955 for residual disease prediction) have further refined resistance stratification and identified key resistance-associated proteins (83–85). Multi-omics clustering has delineated subtypes with differential drug sensitivities (86). **Table 3** summarizes representative multi-omics integration studies. Despite promising results, multi-omics signatures currently remain Tier 3 exploratory biomarkers due to requirements for fresh frozen tissue, high cost, and lack of prospective clinical validation. Standardization of assay platforms and analytical pipelines will be required before clinical implementation.

**Table 3:** Multi-omics and AI-derived biomarker models for PARP inhibitor response prediction.

Study (year)	Modality	Method	Cohort size (Train/Val)	Performance metric	Key PARPi finding	Major limitations	Evidence tier
Kang et al., 2022 (44)	Transcriptomic	tHRD aberrant transcript usage signature	Train n=342; Val n=187	AUC 0.87 for PARPi response (vs 0.69 for genomic HRD)	Identifies HRP patients who derive PARPi benefit; extends PARPi benefit beyond genomic HRD populations	Retrospective design; no prospective interventional validation; signature is platform-dependent	Translational/Tier 3
Wu et al.2025 (83)	Transcriptomic + AI	AIDPI deep learning prognostic index	Train n=521; Val n=268	AUC 0.82 for PFS prediction	Outperforms standard clinical risk factors; MFAP4 identified as a key PARPi resistance mediator	Single-center development; no external validation across independent populations; black-box model interpretability issues	Translational/Tier 3
Zhang et al., 2024 (84)	Single-cell transcriptomics	BeyondCell drug response algorithm	n=42 paired pre/post-PARPi samples	Identified 4 distinct drug-response subtypes	Single-cell resolution of PARPi resistance heterogeneity; identifies targetable resistant subclones.	Small sample size; limited correlation with long-term clinical outcomes; high assay cost	Exploratory/Tier 3
Yasar et al., 2025 (85)	Proteomic	Random forest + SHAP feature selection	Train n=128; Val n=64	AUC 0.955 for PARPi resistance classification	Identified 5 key protein markers of PARPi resistance overlapping with HR repair pathways	Retrospective; FFPE staining variability across centers; no prospective clinical utility data	Translational/Tier 3
Kim et al., 2026 (86)	Multi-omics integration	Transcriptomic + proteomic network analysis	n=89 paired pre/post-treatment samples	Immune signature AUC 0.79 for recurrence prediction	IL7R/IRF8/PTPRC immune signature associated with post-PARPi recurrence; reveals immune microenvironment shifts	Small discovery cohort; no functional validation of targets; assay not clinically available	Exploratory/Tier 3

PARPi, poly (ADP-ribose) polymerase inhibitor; HRD, homologous recombination deficiency; HRP, homologous recombination proficient; tHRD, transcriptional HRD; AUC, area under the curve; PFS, progression-free survival; AI, artificial intelligence; AIDPI, AI-derived prognostic index; SHAP, SHapley Additive exPlanations; FFPE, formalin-fixed paraffin-embedded; MFAP4, microfibril-associated protein 4; Train, training cohort; Val, validation cohort.

### 7.2 AI-based histopathological and multimodal prediction

The core value of AI in PARPi resistance prediction lies in multimodal data integration—fusing histopathological, genomic, transcriptomic, and proteomic data into a unified predictive framework. Bergstrom et al.’s DeepHRD represents a landmark achievement in AI-based HRD status prediction (87). Trained on breast (n=1,008) and ovarian (n=459) cancer H&E-stained slides, the platform demonstrated in external validation that DeepHRD-predicted HRD samples had a 3.7-fold prolonged platinum-based PFS (14.4 vs. 3.9 months) with an HR of 0.45. Through transfer learning to HGSOC, DeepHRD-predicted HRD samples showed superior OS following first-line platinum therapy (HR 0.46–0.49). The most critical finding is that routine histopathological slides harbor previously underutilized HRD-relevant information extractable by AI,Multiresolution spatial mapping revealed that DeepHRD’s predictions were driven by necrotic-neoplastic tissue enrichment, higher macrophage density, and high-density inflammatory tissue—morphological features not captured by genomic HRD assays. This explains why DeepHRD identified 1.8- to 3.1-fold more HRD patients than molecular testing, suggesting that molecular assays may underestimate the population that truly benefits from PARPi.

Beyond predicting HRD status, a pivotal advance is directly estimating individual treatment benefit. Frenel et al. (88) developed DiaThera, a treatment-aware deep learning model trained on diagnostic H&E slides from the phase III PAOLA-1 trial, which computes an Estimated Treatment Improvement (ETI) score by simulating each patient’s survival risk under both olaparib and placebo conditions. In 421 patients, the ETI score demonstrated a strong treatment-biomarker interaction (interaction HR 0.36, 95% CI 0.22-0.59; p<0.005), comparable to HRD status (interaction HR 0.41, 95% CI 0.25-0.64; p<0.005). Critically, ETI stratified benefit within both HRD-positive and HRD-negative subgroups, identifying potential non-responders among HRD-positive patients and, conversely, HRD-negative patients with predicted benefit who would be excluded under current molecular criteria. The model’s predictions were driven by multifocal tumor-infiltrating lymphocytes, aligning with the known immunomodulatory effects of PARP inhibition. Regarding clinical interpretability, Laios et al. (89) applied XGBoost with SHAP to predict R0 resection (AUC 0.866), identifying key factors influencing surgical outcomes relevant to PARPi maintenance eligibility.

Important limitations of AI models must be acknowledged before clinical translation. First, all published models to date are trained and validated retrospectively, with no prospective interventional trials demonstrating that AI-guided therapy improves patient outcomes (90). Second, model performance is highly sensitive to variations in H&E staining protocols, slide preparation, and scanner types across institutions, and external validation in geographically diverse cohorts remains limited for most models. Third, the “black box” nature of deep learning models raises regulatory and clinical trust issues, with explainable AI approaches still in early development (91). As such, AI-based predictors currently remain Tier 3 exploratory biomarkers, though they represent one of the most promising future directions for the field.

In summary, multi-omics and AI integration represents the highest tier in the PARPi resistance assessment paradigm. Nevertheless, current research in this domain remains predominantly retrospective and single-center, and the generalizability and clinical interpretability of these models require validation through large-scale prospective cohorts. Establishing standardized multi-omics databases and developing interpretable AI decision-support systems will be critical directions for translating this tier into clinical practice.

## 8 A whole-disease-course dynamic decision framework

The determinants of PARP inhibitor efficacy evolve continuously—from the intrinsic HR status at diagnosis, through clonal selection under therapeutic pressure, to the molecular landscape at progression. Emerging functional biomarkers—RAD51 immunohistochemistry, KELIM kinetic modeling, ctDNA dynamics, and immune microenvironment profiling—each capture a distinct dimension of tumor biology that conventional *BRCA*/HRD genotyping cannot resolve. No single biomarker, however, suffices for the sequential decisions required across the entire disease trajectory. The paradigm shift therefore lies in integrating these tools into a longitudinal management framework that dynamically pairs each clinical decision node with the most informative biomarker combination.

### 8.1 Biomarker integration logic across the disease continuum

To embed biomarkers into a clinical workflow, three questions must be answered for each tool: when should it be measured, what specific decision does it inform, and how does its information complement that of other assays? **Table 4** summarizes the temporal characteristics and decision-oriented value of the five biomarker categories evaluated in this review.

**Table 4:** Comparative summary of PARP inhibitor predictive biomarkers.

Biomarker	Sample type	Optimal testing time point	Clinical cutoff	Evidence tier	Key advantages	Key limitations
KELIM score	Standard serum CA-125 (no additional testing)	End of neoadjuvant/adjuvant chemotherapy	≥1.0 = chemosensitive; <1.0 = chemoresistant	Tier 1 (clinically actionable)	No additional assay/specimen cost; evaluated in >10,000 patients; predicts both platinum and PARPi benefit	Indirect functional marker; non-calculable in ~15% of patients; not a regulated diagnostic assay; unassessable in CA-125 non-secretor tumors
RAD51 foci IHC	FFPE tumor tissue	Baseline surgery; post-chemotherapy; at progression	<10-50% RAD51-positive cells = HR deficiency (non-functional HR; cutoff varies by assay)	Tier 2 (clinically promising)	Direct functional readout of current HR capacity; dynamic with treatment; predicts PARPi benefit independent of genomics.	Scoring heterogeneity across centers; requires standardized pathology training; cutoff values not yet globally harmonized.
Tissue HRD (GIS)	Archival FFPE tumor tissue	At initial diagnosis	≥33-42 = HRD; 33-50 = gray zone	Tier 1 (regulatory approved)	Regulatory approved for PARPi indication; widely available in high-resource settings	Static marker; does not capture dynamic HR restoration; high cost; assay fails in 20-25% of insufficient samples
ctDNA MRD monitoring	Serial plasma samples	Pre-maintenance; every 3 months during maintenance	Detectable tumor-specific mutations = MRD positive	Tier 2 (clinically promising)	Non-invasive; enables serial dynamic monitoring; detects resistance 4 months before radiographic progression.	High cost; ~20% false negative rate for small-volume disease; CHIP-related false positives require tumor-informed design
ctDNA resistance genotyping	Plasma at progression	At time of PARPi resistance	Any detectable reversion mutation = HR-restored resistance	Tier 2 (clinically actionable)	Non-invasive resistance genotyping; guides selection of subsequent therapy	Does not detect non-genetic resistance mechanisms; lower sensitivity in low tumor fraction disease
Multi-omics/AI signatures	Tissue/plasma	Exploratory use only	Variable by signature	Tier 3 (exploratory)	High predictive accuracy in retrospective cohorts; captures complex tumor biology	No prospective clinical validation; poor external generalizability; high cost; black-box model interpretability issues
Peripheral immune biomarkers	Peripheral blood/tumor tissue	Baseline; on-treatment	Variable by assay	Tier 3 (exploratory)	Predicts benefit from PARPi + immunotherapy combinations	Poor inter-assay reproducibility; no validated clinical cutoffs; not PARPi-specific

PARPi, poly(ADP-ribose) polymerase inhibitor; KELIM, CA-125 elimination rate constant; CA-125, cancer antigen 125; IHC, immunohistochemistry; FFPE, formalin-fixed paraffin-embedded; HRD, homologous recombination deficiency; HR, homologous recombination; GIS, genomic instability score; ctDNA, circulating tumor DNA; MRD, minimal residual disease; CHIP, clonal hematopoiesis of indeterminate potential; AI, artificial intelligence.

These tools differ fundamentally in their temporal relevance: genotyping defines the starting label, functional and pharmacodynamic assays provide dynamic weighting at the pivotal maintenance decision, and liquid biopsy with immune monitoring enable longitudinal surveillance. Accordingly, they cannot be deployed as a single baseline panel but must be orchestrated along a predefined, phase-specific integration pathway.

The whole disease course can be distilled into four critical decision nodes, each calling upon a distinct biomarker combination. **Table 5** provides a concise overview of this node-based integration framework, with key considerations and supporting evidence summarized for each node. This four-node structure transforms clinical management from a single time-point decision into a sequence of dynamic, biomarker-informed assessments.

**Table 5:** Clinical decision framework for functional biomarker-guided PARP inhibitor therapy across the disease course.

Decision node	Disease phase	Current standard of care	Proposed biomarker panel (priority)	Result interpretation	Proposed action (hypothesis for testing)	Evidence level
Node 1: Baseline stratification	At diagnosis/post-primary cytoreductive surgery	One-time static genotyping: germline + somatic BRCA1/2 and tissue HRD (GIS) testing performed once on baseline tumor tissue to assign a binary PARPi-eligible/not-eligible label. No functional HR-capacity assay (RAD51 IHC), chemosensitivity kinetics (KELIM), or immune profiling. HRD status is treated as fixed for the entire disease course.	Standard of care: BRCA1/2 + tissue HRD (GIS); Proposed: RAD51 IHC; Exploratory: peripheral immune markers	BRCA/HRD+ = regulatory PARPi indication; RAD51-low = HR deficiency (non-functional HR; HR pathway inactive); RAD51-high = HR proficiency (functional HR)	Platinum-based chemotherapy followed by PARPi maintenance remains the current standard for eligible patients; RAD51-high HRD+ patients could be evaluated for PARPi plus anti-angiogenic combination	Tier 1 (BRCA/HRD); Tier 2 (RAD51)
Node 2: Maintenance initiation	Post-chemotherapy (before PARPi start)	Radiographic (CT/MRI) + CA-125 response assessment only. PARPi maintenance is offered uniformly to every BRCA/HRD-eligible patient regardless of chemosensitivity, MRD status, or functional HR recovery. No quantitative benefit stratification; PARPi monotherapy vs. PARPi+bevacizumab is not biomarker-guided.	Core: KELIM score; Optional: ctDNA MRD; Adjunct: post-chemotherapy RAD51 IHC	KELIM >=1.0 + ctDNA- = high PARPi benefit; KELIM <1.0 + ctDNA+ = low benefit/high primary resistance risk	KELIM ≥1.0 could support standard PARPi monotherapy; KELIM <1.0 could prompt evaluation of PARPi plus bevacizumab; ctDNA+ patients could be considered for early therapy escalation or clinical trials	Tier 1 (KELIM); Tier 2 (ctDNA MRD)
Node 3: On-treatment monitoring	During PARPi maintenance (every 3 months)	CA-125 + clinical assessment every 3 months; imaging only when symptoms suggest progression or on routine annual review. No serial molecular monitoring for resistance. PARPi continues unchanged until radiographic progression, by which time resistant clones are already established—no repeat biopsy on asymptomatic molecular relapse.	Routine: CA-125 + KELIM-PARP; Investigational: serial ctDNA MRD	3-month ctDNA clearance = good response; persistent/rising ctDNA = early resistance; declining KELIM = reduced PARPi sensitivity	ctDNA clearance could support continuing standard PARPi for the indicated duration; rising or persistent ctDNA could prompt re-biopsy and evaluation of therapy escalation (ATRi/anti-angiogenic/immunotherapy)	Tier 2 (ctDNA dynamics); Tier 3 (KELIM-PARP)
Node 4: Progression	At radiographic/clinical PARPi progression	Empiric switch to next-line chemotherapy (e.g., pegylated liposomal doxorubicin, gemcitabine, topotecan) +/- bevacizumab based on clinical judgment. No standardized resistance workup: routine ctDNA reversion-profiling and repeat RAD51 IHC are not performed; HR-restored vs. non-HR-mediated resistance is not distinguished.	Proposed: ctDNA resistance mutation profiling; If accessible: re-biopsy for RAD51 IHC	Reversion mutation + RAD51-high = HR-restored resistance; RAD51-low = non-HR-mediated resistance	HR-restored resistance could support discontinuing PARPi and evaluating non-PARPi therapy or ATRi combination trials; RAD51-low patients could be considered for PARPi rechallenge or alternative combination strategies	Tier 2 (ctDNA genotyping); Tier 2 (RAD51 re-biopsy)

PARPi, poly(ADP-ribose) polymerase inhibitor; HRD, homologous recombination deficiency; GIS, genomic instability score; IHC, immunohistochemistry; KELIM, CA-125 elimination rate constant; CA-125, cancer antigen 125; ctDNA, circulating tumor DNA; MRD, minimal residual disease; ATRi, ATR inhibitor; KELIM-PARP, KELIM score adapted for PARP inhibitor monitoring.

### 8.2 Clinical decision pathway by node

As a hypothesis-generating framework, we outline below how each biomarker could be considered at each decision node under different resource settings. These are conceptual proposals for prospective evaluation, not clinical recommendations:

(1) Node 1: Initial diagnosis and post-surgery

All patients, all resource settings: Germline and somatic BRCA1/2 testing + HRD testing (Tier 1 standard of care) + KELIM calculation (Tier 1, no-additional-assay-cost). Reference center settings: RAD51 IHC could be added, particularly for patients with equivocal HRD scores (33–50 gray zone) or BRCA mutations with clinical features suggestive of HR proficiency. Hypothesis for testing: unfavorable KELIM combined with RAD51-High status could identify patients in whom PARPi plus bevacizumab maintenance might be evaluated instead of PARPi monotherapy, including within HRD/BRCA-mutant populations.

(2) Node 2: Post-chemotherapy, maintenance therapy initiation

All patients, all resource settings: Repeat CA-125 for KELIM confirmation; standard clinical and radiologic assessment. Reference center settings: ctDNA MRD testing could be considered. Hypothesis for testing: ctDNA MRD-positive patients appear to derive the greatest absolute benefit from PARPi maintenance and could be prioritized in future validation studies; MRD-negative patients have favorable outcomes regardless of maintenance strategy and may be candidates for de-escalation in clinical trials

(3) Node 3: Maintenance therapy surveillance

All patients, all resource settings: 3-monthly CA-125 and clinical assessment per standard of care. Reference center settings: Serial ctDNA monitoring every 3–6 months could be explored for high-risk patients (MRD positive post-chemotherapy, BRCA wild type). Hypothesis for testing: a rising ctDNA allele fraction or detection of BRCA reversion mutations may precede radiographic progression by 3–6 months; in the setting of rising ctDNA without radiologic progression, this could prompt enrollment in early intervention clinical trials or evaluation of combination therapy rather than continued PARPi monotherapy

(4) Node 4: Radiographic progression

All patients, all resource settings: Repeat germline BRCA testing if not previously done; clinical assessment for subsequent therapy. Reference center settings: Metastatic biopsy for repeat RAD51 IHC + ctDNA profiling for resistance mechanisms (reversion mutations, methylation loss, etc.) could be considered. Hypothesis for testing: RAD51-High status or detection of BRCA reversion mutations may indicate HR function restoration; suggesting limited benefit from further PARPi monotherapy; such patients could be evaluated for non-PARPi-containing regimens (e.g., pegylated liposomal doxorubicin, bevacizumab, immunotherapy combinations, ATR inhibitors within clinical trials)

**Figure 1** translates this node-based integration framework into a five-stage clinical pathway spanning diagnosis to post-progression management. The framework represents a conceptual roadmap grounded in the evidence reviewed in preceding sections, rather than a validated clinical algorithm. While each biomarker has been independently associated with PARP inhibitor outcomes, their combined, sequential deployment has not been prospectively tested in interventional trials. The pathways depicted in **Figure 1** therefore serve as testable hypotheses that warrant evaluation in future biomarker-integrated clinical trials. By deploying each tool at the time point where its information content is maximal—genotype at diagnosis, pharmacodynamics at the maintenance decision, liquid biopsy for surveillance, and functional re-assessment at progression—this model illustrates how PARP inhibitor therapy could evolve from a static, eligibility-based paradigm toward a dynamic, node-driven precision strategy. Accordingly, the node-based proposals above should not be interpreted as an established treatment-selection strategy or as formal guidance; they remain hypotheses pending prospective validation.

**Figure 1:**
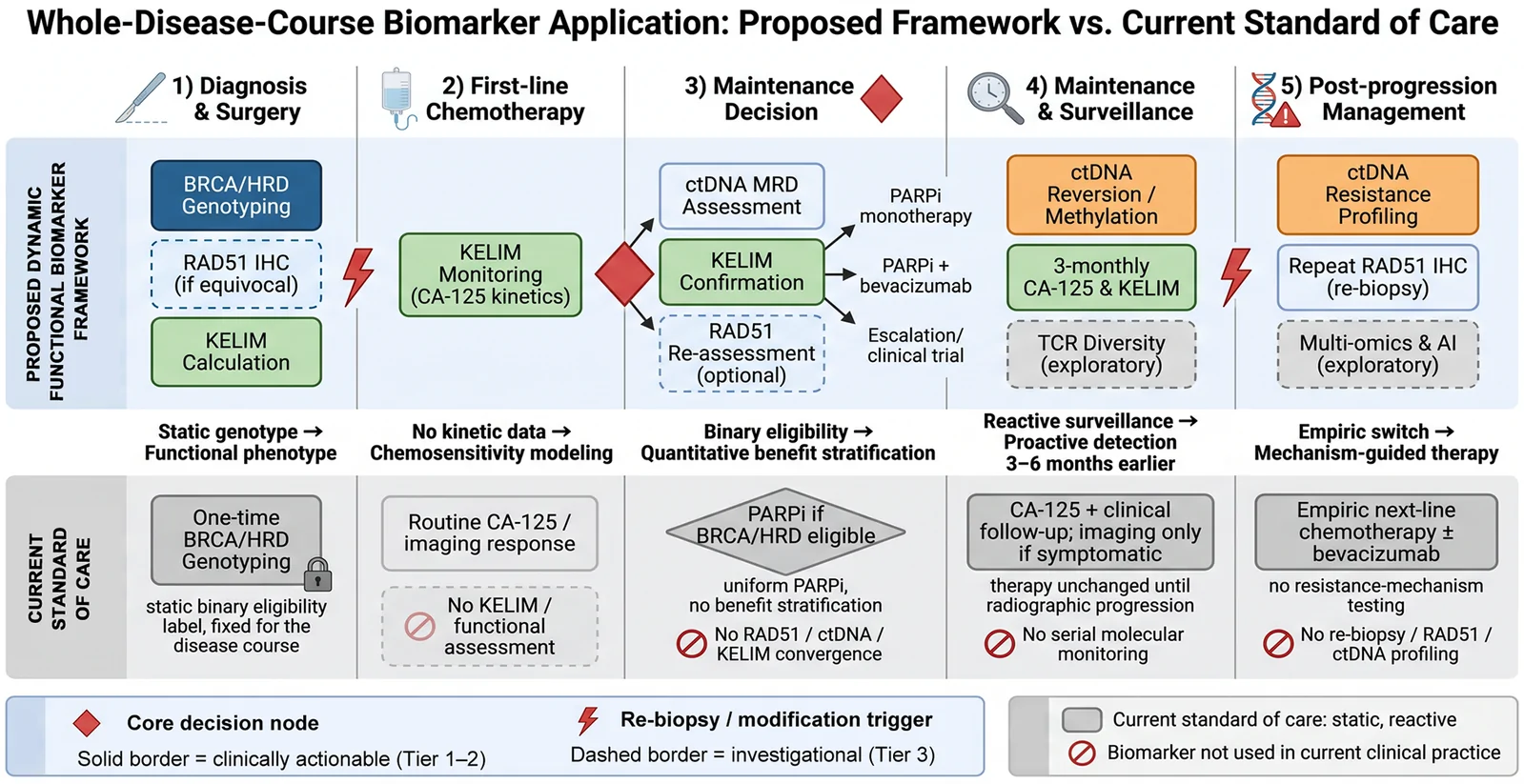
Whole-disease-course biomarker application: the proposed dynamic functional biomarker framework compared with current standard of care. The upper (color-coded) lane depicts the sequential deployment of functional biomarkers across five clinical stages of high-grade serous ovarian cancer management: (1) diagnosis and surgery, (2) first-line chemotherapy, (3) the maintenance treatment decision, (4) maintenance surveillance, and (5) post-progression management. Box colors denote assay class and evidence tier: dark blue, Tier 1 tissue-based assays (BRCA/HRD genotyping); light blue, Tier 2 tissue/liquid assays (RAD51 immunohistochemistry, ctDNA minimal residual disease); green, serum KELIM kinetic monitoring; orange, Tier 2 liquid biopsy (serial ctDNA); and grey dashed boxes, Tier 3 exploratory immune and artificial intelligence-based assays. Solid borders indicate clinically actionable assays (Tier 1–2) and dashed borders indicate investigational assays (Tier 3). At the core maintenance decision node (red diamond), KELIM, RAD51, and ctDNA MRD results converge in the proposed framework to inform hypothetical choices among PARP inhibitor (PARPi) monotherapy, PARPi plus bevacizumab, and escalation/clinical-trial enrollment; red lightning bolts mark potential triggers for repeat biopsy or therapeutic modification. The lower (grayscale) lane depicts current standard of care aligned stage by stage—a single static BRCA/HRD genotype assigned once at diagnosis, uniform PARPi administration without functional stratification, CA-125/imaging-only surveillance that defers treatment change until radiographic progression, and an empiric switch to subsequent chemotherapy without resistance-mechanism testing. Red prohibition symbols indicate biomarkers not used in current routine practice. The intervening tags summarize the principal shifts introduced by the framework: from static genotype to functional phenotype, from absent kinetic data to chemosensitivity modeling, from binary eligibility to quantitative benefit stratification, from reactive to proactive pre-radiographic surveillance, and from empiric to mechanism-guided post-progression therapy. PARPi, poly (ADP-ribose) polymerase inhibitor; HRD, homologous recombination deficiency; IHC, immunohistochemistry; KELIM, CA-125 elimination rate constant; ctDNA, circulating tumor DNA; MRD, minimal residual disease; TCR, T-cell receptor; AI, artificial intelligence.

## 9 Challenges, health economics, future directions, and critical view

### 9.1 Standardization and evidence gaps

Functional biomarkers offer a tangible pathway beyond static BRCA/HRD genotyping, yet their integration into routine practice faces interconnected hurdles spanning standardization, accessibility, evidence generation, and data integration. This narrative review has systematically evaluated tissue-based functional HR assessment (RAD51 IHC, functional HRD scoring), serum-based chemosensitivity modeling (KELIM), liquid biopsy strategies (ctDNA reversion, methylation, genomic instability, and MRD monitoring), immune microenvironment biomarkers, and multi-omics/AI integration, covering the literature up to June 2026. To our knowledge, it is the first review to integrate these functional biomarkers into a unified, whole-disease-course decision framework that accounts for evidence level and global resource stratification, thereby distinguishing itself from previous genotype-centric or single-biomarker-focused overviews.

The heterogeneity in RAD51 IHC interpretation—ranging from divergent foci thresholds (≥4 vs ≥5) to varying positivity cutoffs (>10% vs >15%)—persists across studies (17, 18, 26, 30, 92, 93), impeding cross-study comparison and regulatory qualification. Inter-assay concordance remains suboptimal for HRD, KELIM, and ctDNA platforms (50, 68, 94, 95). Resolution requires international consensus guidelines that unify scoring criteria, standardize computational tools, and define minimum performance specifications, complemented by AI-assisted automated image analysis to minimize inter-observer variability. More fundamentally, the evidentiary hierarchy remains a critical barrier. Most functional biomarker studies remain retrospective or *post hoc*; prospective biomarker-stratified randomized trials are essential to establish whether biomarker-guided therapy improves outcomes over empirical treatment (96). Whether biomarker-guided therapy selection improves clinical outcomes—the pivotal transition from prognostic to predictive utility—remains unestablished. This gap requires biomarker-stratified randomized trials or adaptive enrichment umbrella designs (97, 98) that directly address whether functional biomarker-guided therapy outperforms empirical treatment. AI-based multimodal integration promises to manage data complexity, but interpretability and external validation remain prerequisites for clinical adoption (81, 99).

### 9.2 Health economics and global implementation

Health economics and global accessibility must be central to implementation, not an afterthought. Biomarker costs vary by more than 100-fold: KELIM requires no additional testing beyond standard CA-125 monitoring; RAD51 IHC costs approximately $100–200 per sample (34); tissue HRD testing costs $2000–4000; ctDNA MRD and multi-omics testing cost $3000–5000 per assay (100). For low- and middle-income countries (LMICs) where 70% of global ovarian cancer deaths occur, high-cost biomarkers will remain inaccessible for the foreseeable future. “Reference centers” denotes regional or national cancer hospitals with multidisciplinary gynecologic oncology expertise, accredited pathology and molecular laboratories, and quality-assured immunohistochemistry and liquid-biopsy workflows; through specimen referral and telepathology networks they serve as hubs for surrounding lower-resource centers, and such centers already operate within many low- and middle-income countries. A tiered implementation strategy could therefore be evaluated: KELIM could be adopted first as a functional biomarker across all settings; RAD51 IHC could be prioritized for regional reference centers; and high-cost ctDNA, multi-omics, and AI assays could be reserved for high-resource settings and clinical trials. If validated, such a tiered approach could extend functional biomarker benefits beyond high-income countries and improve outcomes globally. Finally, real-world validation is needed to confirm functional biomarker value. Real-world disparities, such as older patients being less likely to receive PARP inhibitors, may influence biomarker performance (101), and establishing functional biomarker-driven registry studies would validate utility across broad populations while creating a “practice-data-evidence-optimization” learning healthcare system (102).

### 9.3 Operational feasibility across the disease course

Beyond analytical performance and cost, practical deployment depends on whether the required specimen is obtainable at each stage and whether results return in time to influence treatment. These stage-specific constraints are summarized in **Supplementary Table S1**, complementing the node-specific recommendations in Section 8.2 and the assay characteristics in **Table 4**.

Three principles apply. First, procedural burden varies by stage and should guide assay choice: at diagnosis the scheduled surgery already provides tissue, allowing BRCA/HRD and RAD51 to share one formalin-fixed, paraffin-embedded block (26); post-chemotherapy tissue is usually unavailable; and at progression invasive biopsy is often precluded by lesion inaccessibility or reduced patient fitness, making plasma ctDNA the practical first-line test, with tissue reflexed only when liquid profiling is non-informative (69, 71, 72). Second, assay failure requires stage-specific contingencies—HRD testing fails in 10–30% of inadequate samples (26), for which RAD51 on the same block offers a functional fallback; KELIM is non-calculable in approximately 15% of patients (103), where radiologic and clinical assessment substitutes; and ctDNA negativity does not exclude small-volume disease (~20% false-negative rate) or replace imaging (71, 72). Third, turnaround times must fit the relevant treatment window, supported by standardized pre-analytical handling (plasma collection in cell-stabilization or EDTA tubes with prompt processing), trained personnel, and reimbursement pathways (104); regional reference laboratories can serve as hubs that operationalize the tiered strategy above (100)—KELIM universally, RAD51 at reference centers, and ctDNA/multi-omics in high-resource settings and clinical trials.

From a critical perspective, we contend that the paradigm shift from static genotype eligibility to dynamic functional phenotyping is increasingly feasible with tools already in clinical use or late-stage validation. The KELIM score alone could immediately narrow the eligibility–benefit gap in resource-limited settings. Once standardized, RAD51 IHC offers the most direct functional readout and can resolve ambiguous HRD gray zones. Serial ctDNA monitoring represents the future of real-time resistance detection. No single functional biomarker is sufficient; their sequential, complementary deployment across the disease course is essential. Our integrated framework is intended to guide future prospective trial design and stimulate health-economic analyses to justify tiered global implementation. Consistent with the purpose of a narrative review, these frameworks are offered as testable concepts to guide future investigation, not as high-level clinical evidence or formal guidelines. By explicitly addressing the eligibility–benefit gap and proposing a practical, node-based deployment model, this review moves beyond describing biomarkers to providing a clinically actionable roadmap that, once validated, could advance adaptive precision oncology and ultimately close the gap between PARP inhibitor eligibility and true patient benefit.

## 10 Conclusion

BRCA/HRD testing established the foundation for precision PARP inhibitor therapy, but its inherent limitations—the eligibility–benefit gap, inability to capture clonal evolution, and negligible predictive value in HRP tumors—demand a shift from static genotyping to dynamic functional phenotyping.

The multi-node model proposed here (diagnosis → post-chemotherapy → maintenance → progression) enables adaptive, longitudinal decision-making. At maintenance initiation, RAD51 IHC and KELIM refine treatment selection beyond BRCA/HRD status. During surveillance, ctDNA dynamics and immune monitoring provide early warning of resistance before radiographic progression. At overt progression, re-biopsy, RAD51, and ctDNA profiling elucidate the underlying resistance mechanism. This sequential deployment narrows the eligibility–benefit gap by directing functionally HR-deficient patients toward timely PARP inhibitor maintenance while sparing those with restored HR function.

As a framework for prospective evaluation, we propose that KELIM could be calculated during first-line chemotherapy in all settings as an adjunct to BRCA/HRD testing, that RAD51 IHC be used in reference centers to resolve equivocal HRD scores and re-assess HR status at progression, that ctDNA testing for BRCA reversion and MRD be considered for high-risk patients where available without replacing standard monitoring, and that immune, multi-omics, and AI-based biomarkers remain investigational and restricted to clinical trials. These proposals require prospective validation before any adoption into routine care. Functional biomarkers enable a precision paradigm in which therapy is dynamically guided by real-time tumor biology. The central question is no longer “Does this patient carry a BRCA mutation?” but “Is this tumor functionally HR-deficient now, and how is it evolving?” Closing the eligibility–benefit gap depends not on refining gene panels alone, but on embracing the dynamic biology they only partially encode.
