# Supplementary material for: Functional biomarkers complementing static BRCA/HRD testing for dynamic PARP inhibitor therapy in ovarian cancer: a narrative review
Source: Front Oncol. 2026 Oct 13;16:1989686. doi: 10.3389/fonc.2026.1989686 (PMC13711281; doi:10.3389/fonc.2026.1989686)
Supplement: Supplementary file 1 [file Supplementaryfile1.docx]

**Supplementary Methods: Detailed Literature Search Strategy**

**Version 2.0 — updated search (September 22, 2026); original search: June 20, 2026.**

Search date: September 22, 2026 (updated search; original search performed on June 20, 2026)

Databases: PubMed (MEDLINE), Embase (Elsevier), and Web of Science Core Collection (Clarivate)

Coverage: from database inception to September 22, 2026 (updated search)

Limits: human studies; English-language full-text articles

Structure: terms within each domain were combined with OR, and the three domains (ovarian cancer; PARP inhibitors and biomarkers; precision medicine) were combined with AND.

**Rationale for class-level and agent-specific terms**

The PARP-inhibitor domain combined the controlled-vocabulary heading for the class (MeSH, Emtree) with free-text class terms ("PARP inhibitor*", PARPi). These class-level terms retrieve records for every agent in the class, so the China-approved PARP inhibitors (fuzuloparib/fluzoparib, pamiparib, senaparib) and next-generation PARP1-selective inhibitors were already captured regardless of whether each generic name was enumerated; their absence from the original agent list therefore did not exclude any records. For full transparency the agent-specific list has nevertheless been expanded to include the China-approved agents, their development codes, and next-generation agents (saruparib/AZD5305, AZD9574, NMS-03305293), together with relevant brand names.

Note: This is Version 2.0 of the search strategy. The updated, expanded search was performed on September 22, 2026 in response to reviewer comments; it did not identify any additional studies that altered the evidence or conclusions of the review, and no changes to the manuscript content were required.

**1. PubMed (MEDLINE) search strategy**

| **Search ID** | **Search query** |
| --- | --- |
| #1 | "Ovarian Neoplasms"[MeSH Terms] OR "Fallopian Tube Neoplasms"[MeSH Terms] OR "Peritoneal Neoplasms"[MeSH Terms] |
| #2 | ovarian neoplasm*[Title/Abstract] OR ovarian cancer[Title/Abstract] OR ovarian carcinoma[Title/Abstract] OR high-grade serous ovarian cancer[Title/Abstract] OR HGSOC[Title/Abstract] OR fallopian tube cancer[Title/Abstract] OR primary peritoneal cancer[Title/Abstract] OR epithelial ovarian cancer[Title/Abstract] OR EOC[Title/Abstract] |
| #3 | #1 OR #2 |
| #4 | "Poly(ADP-ribose) Polymerase Inhibitors"[MeSH Terms] |
| #5 | PARP inhibitor*[Title/Abstract] OR PARPi[Title/Abstract] OR PARP1 inhibitor*[Title/Abstract] OR olaparib[Title/Abstract] OR niraparib[Title/Abstract] OR rucaparib[Title/Abstract] OR veliparib[Title/Abstract] OR talazoparib[Title/Abstract] OR fluzoparib[Title/Abstract] OR fuzuloparib[Title/Abstract] OR pamiparib[Title/Abstract] OR senaparib[Title/Abstract] OR saruparib[Title/Abstract] OR AZD5305[Title/Abstract] OR AZD9574[Title/Abstract] OR NMS-293[Title/Abstract] OR NMS-03305293[Title/Abstract] OR BGB-290[Title/Abstract] OR IMP4297[Title/Abstract] OR SHR3162[Title/Abstract] OR next-generation PARP inhibitor*[Title/Abstract] OR selective PARP1 inhibitor*[Title/Abstract] OR Lynparza[Title/Abstract] OR Zejula[Title/Abstract] OR Rubraca[Title/Abstract] OR Talzenna[Title/Abstract] OR AiRuiYi[Title/Abstract] |
| #6 | RAD51[Title/Abstract] OR KELIM[Title/Abstract] OR CA-125 elimination rate constant[Title/Abstract] OR circulating tumor DNA[Title/Abstract] OR ctDNA[Title/Abstract] OR homologous recombination deficiency[Title/Abstract] OR HRD[Title/Abstract] OR T cell receptor[Title/Abstract] OR TCR[Title/Abstract] OR tumor microenvironment[Title/Abstract] OR artificial intelligence[Title/Abstract] OR deep learning[Title/Abstract] |
| #7 | #4 OR #5 OR #6 |
| #8 | predictive biomarker*[Title/Abstract] OR prognostic biomarker*[Title/Abstract] OR precision medicine[Title/Abstract] OR treatment resistance[Title/Abstract] OR clinical decision-making[Title/Abstract] OR "Precision Medicine"[MeSH Terms] |
| #9 | #3 AND #7 AND #8 |
| #10 | #9 AND "humans"[MeSH Terms] AND English[lang] |
| #11 | #10 AND ("2026/09/22"[PDAT] : "0001/01/01"[PDAT]) |

**2. Embase search strategy (embase.com syntax)**

| **Search ID** | **Search query** |
| --- | --- |
| #1 | 'ovary tumor'/exp OR 'fallopian tube tumor'/exp OR 'peritoneum tumor'/exp |
| #2 | 'ovarian neoplasm*':ti,ab,kw OR 'ovarian cancer':ti,ab,kw OR 'ovarian carcinoma':ti,ab,kw OR 'high-grade serous ovarian cancer':ti,ab,kw OR hgsoc:ti,ab,kw OR 'fallopian tube cancer':ti,ab,kw OR 'primary peritoneal cancer':ti,ab,kw OR 'epithelial ovarian cancer':ti,ab,kw OR eoc:ti,ab,kw |
| #3 | #1 OR #2 |
| #4 | 'poly(ADP-ribose) polymerase inhibitor'/exp |
| #5 | 'PARP inhibitor*':ti,ab,kw OR parpi:ti,ab,kw OR 'PARP1 inhibitor*':ti,ab,kw OR olaparib:ti,ab,kw OR niraparib:ti,ab,kw OR rucaparib:ti,ab,kw OR veliparib:ti,ab,kw OR talazoparib:ti,ab,kw OR fluzoparib:ti,ab,kw OR fuzuloparib:ti,ab,kw OR pamiparib:ti,ab,kw OR senaparib:ti,ab,kw OR saruparib:ti,ab,kw OR azd5305:ti,ab,kw OR azd9574:ti,ab,kw OR nms-293:ti,ab,kw OR nms-03305293:ti,ab,kw OR bgb-290:ti,ab,kw OR imp4297:ti,ab,kw OR shr3162:ti,ab,kw OR 'next-generation PARP inhibitor*':ti,ab,kw OR 'selective PARP1 inhibitor*':ti,ab,kw OR lynparza:ti,ab,kw OR zejula:ti,ab,kw OR rubraca:ti,ab,kw OR talzenna:ti,ab,kw OR airuiyi:ti,ab,kw |
| #6 | rad51:ti,ab,kw OR kelim:ti,ab,kw OR "circulating tumor DNA":ti,ab,kw OR ctdna:ti,ab,kw OR "homologous recombination deficiency":ti,ab,kw OR hrd:ti,ab,kw OR "T cell receptor":ti,ab,kw OR tcr:ti,ab,kw OR "tumor microenvironment":ti,ab,kw OR "artificial intelligence":ti,ab,kw OR "deep learning":ti,ab,kw |
| #7 | #4 OR #5 OR #6 |
| #8 | 'predictive biomarker*':ti,ab,kw OR 'prognostic biomarker*':ti,ab,kw OR 'precision medicine':ti,ab,kw OR 'treatment resistance':ti,ab,kw OR 'clinical decision-making':ti,ab,kw OR 'precision medicine'/exp |
| #9 | #3 AND #7 AND #8 |
| #10 | #9 AND [humans]/lim AND [english]/lim |

**3. Web of Science Core Collection search strategy**

Web of Science does not use controlled headings; all terms are searched by topic (TS = title, abstract, author keywords, and Keywords Plus).

| **Search ID** | **Search query** |
| --- | --- |
| #1 | TS=(ovarian neoplasm* OR ovarian cancer OR ovarian carcinoma OR "high-grade serous ovarian cancer" OR HGSOC OR "fallopian tube cancer" OR "primary peritoneal cancer" OR "epithelial ovarian cancer" OR EOC) |
| #2 | TS=("PARP inhibitor*" OR PARPi OR "PARP1 inhibitor*" OR olaparib OR niraparib OR rucaparib OR veliparib OR talazoparib OR fluzoparib OR fuzuloparib OR pamiparib OR senaparib OR saruparib OR AZD5305 OR AZD9574 OR NMS-293 OR NMS-03305293 OR BGB-290 OR IMP4297 OR SHR3162 OR "next-generation PARP inhibitor*" OR "selective PARP1 inhibitor*" OR Lynparza OR Zejula OR Rubraca OR Talzenna OR AiRuiYi OR RAD51 OR KELIM OR "circulating tumor DNA" OR ctDNA OR "homologous recombination deficiency" OR HRD OR "T cell receptor" OR TCR OR "tumor microenvironment" OR "artificial intelligence" OR "deep learning") |
| #3 | TS=("predictive biomarker*" OR "prognostic biomarker*" OR "precision medicine" OR "treatment resistance" OR "clinical decision-making") |
| #4 | #1 AND #2 AND #3 |
| #5 | #4 refined by: Languages = English |

**4. Additional hand-searching**

Reference lists of key articles and relevant guidelines were hand-searched, and 2025–2026 online-first publications not yet indexed at the time of the search were retrieved directly.
