# Supplementary material for: Functional biomarkers complementing static BRCA/HRD testing for dynamic PARP inhibitor therapy in ovarian cancer: a narrative review
Source: Front Oncol. 2026 Oct 13;16:1989686. doi: 10.3389/fonc.2026.1989686 (PMC13711281; doi:10.3389/fonc.2026.1989686)
Supplement: Supplementary file 2 [file Table1.docx]

**Supplementary Table S1. Operational feasibility of biomarker testing by disease stage.**

*Specimen availability, procedural burden, and stage-specific delivery constraints for the biomarkers recommended at each decision node of the whole-disease-course framework (Section 8, Figure 1; discussed in Section 9.1).*

| **Disease stage / node** | **Readily available specimen** | **Tests requiring no additional procedure** | **Tests requiring only a blood draw** | **Tests requiring invasive biopsy** | **Principal feasibility constraint** |
| --- | --- | --- | --- | --- | --- |
| **Node 1: Diagnosis / surgery** | Fresh tumor from cytoreductive surgery; baseline CA-125 | BRCA/HRD and RAD51 IHC on the same FFPE block; KELIM initiated from routine CA-125 | Germline BRCA (blood or saliva) | None beyond scheduled surgery | HRD assay failure in 10–30% of inadequate samples; RAD51 scoring requires trained pathology |
| **Node 2: Maintenance initiation** | CA-125 series from the first 100 days of chemotherapy | KELIM (computed retrospectively; free online calculator) | ctDNA MRD (single pre-maintenance plasma sample) | Post-chemotherapy RAD51 usually infeasible—no residual tumor tissue | ctDNA requires centralized platforms and is cost-limited; no tissue available for functional re-testing |
| **Node 3: Maintenance surveillance** | Serial CA-125 at routine follow-up visits | CA-125 / KELIM-PARP | Serial ctDNA every 3–6 months (high-risk patients) | Not required | Low peritoneal shedding (~20% false-negative in small-volume disease); CHIP false positives; cost; cannot replace imaging |
| **Node 4: Progression** | Plasma; archival FFPE; germline sample | Repeat germline BRCA if not previously performed | ctDNA resistance (reversion / methylation) profiling | Repeat biopsy for RAD51 IHC (selected cases) | Lesion inaccessibility and reduced patient fitness after multiple therapy lines; plasma-first, with biopsy reserved for accessible or discordant lesions |

**Abbreviations:** PARPi, poly(ADP-ribose) polymerase inhibitor; HRD, homologous recombination deficiency; IHC, immunohistochemistry; FFPE, formalin-fixed paraffin-embedded; KELIM, CA-125 elimination rate constant; KELIM-PARP, KELIM score adapted for PARP inhibitor monitoring; CA-125, cancer antigen 125; ctDNA, circulating tumor DNA; MRD, minimal residual disease; CHIP, clonal hematopoiesis of indeterminate potential.
